# Multi-orbit lunar GNSS constellation design with distant retrograde orbit and Halo orbit combination

**DOI:** 10.1038/s41598-023-37348-x

**Published:** 2023-06-22

**Authors:** K Wang, Kezhao Li, Shuaikang Lv, YingXiang Jiao, Yunyan Shen, Zhe Yue, Keke Xu

**Affiliations:** 1 School of Surveying and Land Information Engineering, Henan Polytechnic University, Jiaozuo, 454000 China; 2 Collaborative Innovation Center of BDS Research Application, Zhengzhou, 450052 China

**Keywords:** Aerospace engineering, Civil engineering

## Abstract

The Moon is the closest natural satellite to mankind, with valuable resources on it, and is an important base station for mankind to enter deep space. How to establish a reasonable lunar Global Navigation Satellite System (GNSS) to provide real-time positioning, navigation, and timing (PNT) services for Moon exploration and development has become a hot topic for many international scholars. Based on the special spatial configuration characteristics of Libration point orbits (LPOs), the coverage capability of Halo orbits and Distant Retrograde Orbit (DRO) in LPOs is discussed and analyzed in detail. It is concluded that the Halo orbit with a period of 8 days has a better coverage effect on the lunar polar regions and the DRO has a more stable coverage effect on the lunar equatorial regions, and the multi-orbital lunar GNSS constellation with the optimized combination of DRO and Halo orbits is proposed by combining the advantages of both. This multi-orbital constellation can make up for the fact that a single type of orbit requires a larger number of satellites to fully cover the Moon, using a smaller number of satellites for the purpose of providing PNT services to the entire lunar surface. We designed simulation experiments to test whether the multi-orbital constellations meet the full lunar surface positioning requirements, and compare the coverage, positioning, and occultation effects of the four constellation designs that pass the test, and finally obtain a set of well-performing lunar GNSS constellations. The results indicate that the multi-orbital lunar GNSS constellation combining DRO and Halo orbits can cover 100% of the Moon surface, provides there are more than 4 visible satellites at any time on the Moon surface, which meets the navigation and positioning requirements, and the Position Dilution of Precision (PDOP) value is stable within 2.0, which can meet the demand for higher precision Moon surface navigation and positioning.

The Moon is the only natural satellite of the Earth, where there exists large amounts of strategic resources such as rare earth elements, and it can also become a base for future space exploration and development. As a result of the Moon's strategic importance, countries worldwide have dedicated significant amounts of financial, material, and human resources to exploring and studying it, leading to an increasing number of lunar exploration missions. In the face of the increasing number of Moon missions, NASA has clearly stated in its lunar exploration plan its intention to establish a precise and reliable lunar navigation system to ensure the safety and success of future missions^1^. However, the Global Navigation Satellite System (GNSS), which is currently used more successfully on Earth, is more difficult to apply on the Moon due to the Earth–Moon distance, and the occultation effect and signal strength need to be considered^2,3^. Therefore, it is necessary to expand GNSS services to the Moon and build a lunar GNSS system to provide continuous and stable PNT service to the cis-lunar space.

Libration points (LPs), which are force equilibrium points in the circular-restricted three-body problem, have important research value due to their special spatial location and mechanical properties and are widely used in the field of lunar relay communication/navigation satellites^4^. In June 2018, the Chinese lunar exploration project Chang'E-IV mission “Magpie Bridge” relay satellite entered the Halo orbit around the Earth–Moon L2 LP, becoming the world's first satellite to operate in the L2 Halo orbit^5^.

For launching satellites on LPOs, the concept of establishing an Earth–Moon space communication link has been proposed some years ago. Farquhar^6^ was the first to propose the placement of satellites in orbit near the L2-point for broadcasting and communication to the lunar backside region. Later, Carpenter^7^ demonstrated the feasibility of orbits near the L2-point with good coverage for circumlunar probes and the establishment of relay communication constellations on LPOs. Weiren^8^ analyzed and designed the overall scheme of the point relay satellite communication mission based on the characteristics and technical difficulties of the L2-point relay satellite mission. Gao^9^ proposed a lunar communication constellation design using a combination of Halo orbit and DRO, which is a five-satellite constellation consisting of three Halo orbit satellites and two DRO satellites that provides 100% continuous coverage of the entire Moon's surface. However, the communication constellation only has a one-fold coverage of the Moon, so it can only be used for real-time communication and not for navigation and positioning.

Hamera^10^ proposed a Global Navigation Satellite System-like (GNSS-like) lunar GNSS constellation, which consists of Halo orbiting satellites near the L1 and L2 points, to provide navigation and position services for lunar probes. Batista^11^ investigated the theory of lunar GNSS constellation design. Christian^12^ proposed the idea of using L1-point and L2-point Halo orbits to implement a lunar GNSS system and gave a constellation design using a combination of southern and northern families of Halo orbits, but the constellation design has poor coverage of the lunar equator and the future users on the lunar surface cannot continuously meet the minimum number of simultaneous observation satellites required for navigation and positioning. Lei Zhang^13^ designed a two-satellite navigation system around the Lagrangian L1 and L2 points in Earth–Moon space and demonstrated that the system can be used for Earth–Moon space probe navigation, but its positioning effect is not enough to be applied to the actual lunar exploration project because there are only two satellites. Pereira^14^ studied the lunar GNSS constellation design and proposed an interim lunar GNSS constellation design, but the constellation was restricted to frozen orbital conditions.

Pasquale identifies a set of optimization parameters for Keplerian orbits in the lunar-satellite two-body problem, proposes a framework for multi-objective optimization, and constructs a constellation that meets the communication and navigation needs of cis-lunar space. However, he does not discuss the “hybrid” orbit composed of Keplerian orbits and LPOs, but treats LPOs as an enhanced part of the Keplerian orbit constellation^15^. Similarly, Zanotti^16^ constructs a lunar GNSS constellation using multi-objective optimization for Keplerian orbits in the lunar-satellite two-body problem, but does not address “hybrid” orbits. Nallapu^17^ uses mixed-integer genetic algorithm solver based on the constraints of orbital stability and synchronization to solve the LPOs-based lunar communication constellation design problem. To summarize these studies, they all use optimization methods to solve the design problem of lunar navigation and communication constellations, mostly based on Keplerian orbits to form navigation constellations or using LPOs to form communication constellations, and no navigation constellations composed of LPOs have been designed. This is due to the fact that LPOs are non-Keplerian orbits, which cannot use the orbital root number to express the satellite's orbiting state, and their only approximate analytical solutions exist under the restricted three-body problem^18^. For solving the problem of designing a lunar navigation constellation consisting of LPOs using an optimization algorithm, it is more complicated than Keplerian orbits. Therefore, in this study, we use the orbit characteristics to perform a grid search of candidate constellations to determine the optimal orbit.

Based on the above research, this paper proposes a multi-orbital lunar GNSS constellation design to address the shortcomings of the above research. The constellation design takes advantage of the better coverage of the lunar polar regions by Halo orbit and the stable coverage of the lunar equatorial regions by DRO, and combines Halo orbit with DRO to achieve the requirement of full-coverage lunar navigation services using a smaller number of satellites.

## Theory and methods

### Circular-restricted three-body problem

**Figure 1:**
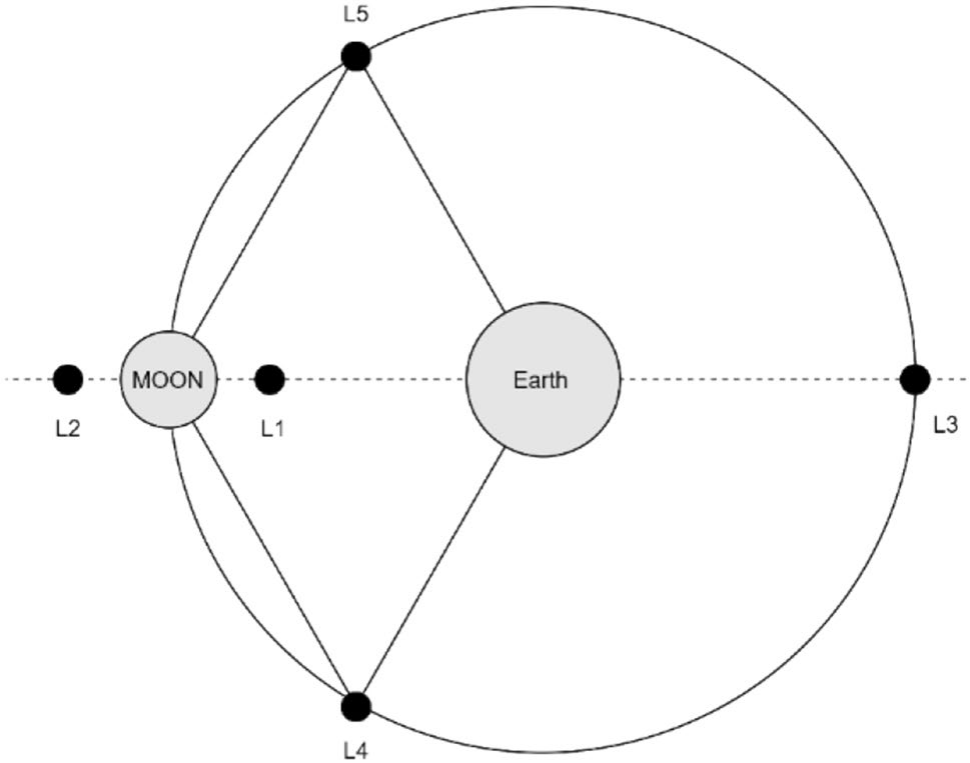
Schematic diagram of Lagrange points.

In 1772, the French scientist Lagrange studied the Earth–Moon system and discovered five special gravitational equilibrium points, called Lagrange points, also known as LPs^19^. As shown in Fig. 1, the translational points in the Earth–Moon system are in gravitational equilibrium, where the points L1, L2, and L3 are co-linear with the Earth–Moon and located in the "weak stability region", i.e., in this region, a small thrust can be used to close the orbit of the spacecraft around the unstable points^20^. The L1-point and L2-point are on both sides of the Moon, and their surrounding orbits have good coverage of the moon surface. Using the special location of the L1 and L2 points, the orbiting satellites around them can be used as Moon orbit relay satellites to provide navigation/communication services to the moon probes^21^.

**Figure 2:**
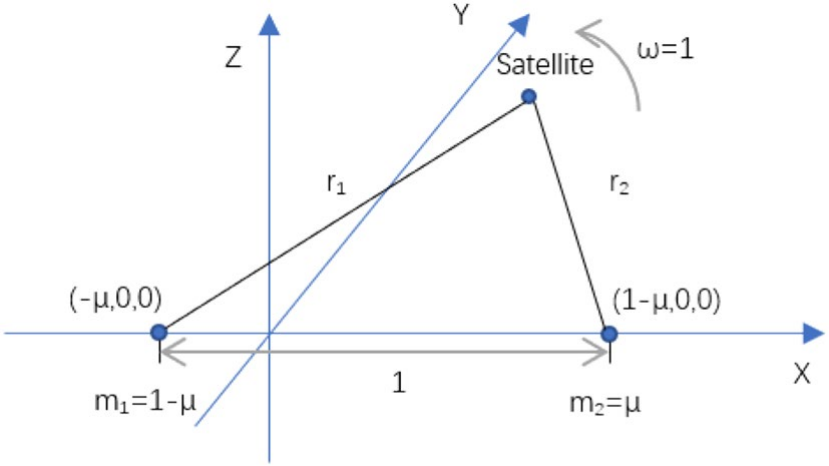
Earth–Moon center of mass convergence coordinate system.

For the Earth–Moon-Libration point orbiting satellite three-body system, it can be considered as a circular-restricted three-body problem (CRTBP) in order to simplify the analytical process of the satellite dynamics model. In the CRTBP problem, let the masses of the Earth and the Moon be $m_{1}$ and $m_{2}$, respectively, and they move in a strictly circular motion around the masses of the two planets. The satellite is affected by the common gravitational force of the Earth and the Moon and is considered as a mass point with negligible mass of its own. As shown in Fig. 2, the coordinate system used for CRTBP is the Earth–Moon center-of-mass conjunction coordinate system, where the origin of the coordinate system is the common center of mass of the Earth and the Moon, the Earth–Moon line is the x-axis, the steering of the Earth and the Moon is the y-axis, and the z-axis forms a plane coordinate system with the x-axis and y-axis.

**Figure 3:**
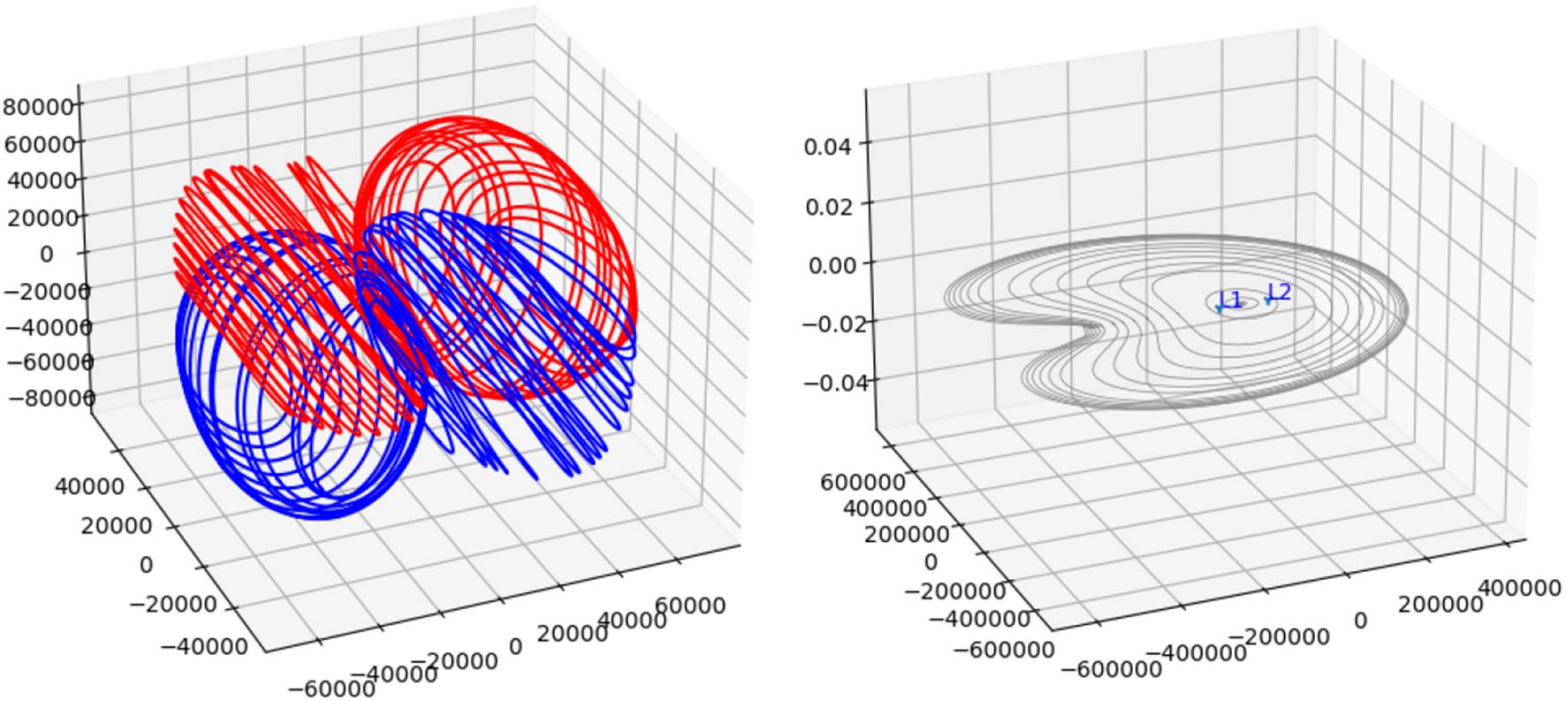
Halo orbit spatial configuration (left) and DRO spatial configuration (right).

In CRTBP, there are five LPs. There are many libration point orbits (LPOs) around these LPs, and there are two types of LPOs: periodic and quasi-periodic. In this paper, we focus on the Moon coverage capability of satellites in periodic orbits and do not consider quasi-periodic orbits for the time being. The periodic orbits around the Earth–Moon L1 and L2 points mainly include Halo orbit, DROs, etc. Halo orbits revolve around the L1 or L2 points, and satellites in Halo orbits rotate around the LP, maintaining roughly equal distance from the Earth and Moon. The angular velocity of the satellites is the same as that of the Earth and Moon, ensuring the satellites to maintain a relatively stationary position, always facing towards the Earth and Moon. Due to the symmetry of the CRTBP, each Halo orbit is surrounded by a Northern and Southern family, and the two Halo orbit families are symmetric about the x–y plane (see Fig. 3 left). Halo orbits depart from the L1 or L2 point and gradually approach the Moon, with their orbital period also decreasing gradually. The DRO is a stable circular retrograde orbit, in which the satellites running around the L1 and L2 points in a direction opposite to that of the Moon around the Earth. The orbital period of the DRO orbit increases with the distance from the Moon. DRO contains two types of space DRO and planar DRO, where the planar DRO runs on the Moon's orbital plane and has better coverage of the equatorial region of the Moon (see Fig. 3, right). In this paper, we take advantage of the good coverage capability of Halo orbit and planar DRO to the Moon, and form them into a multi-orbital constellation to provide stable PNT services to the Moon.

### Coverage capacity analysis

**Figure 4:**
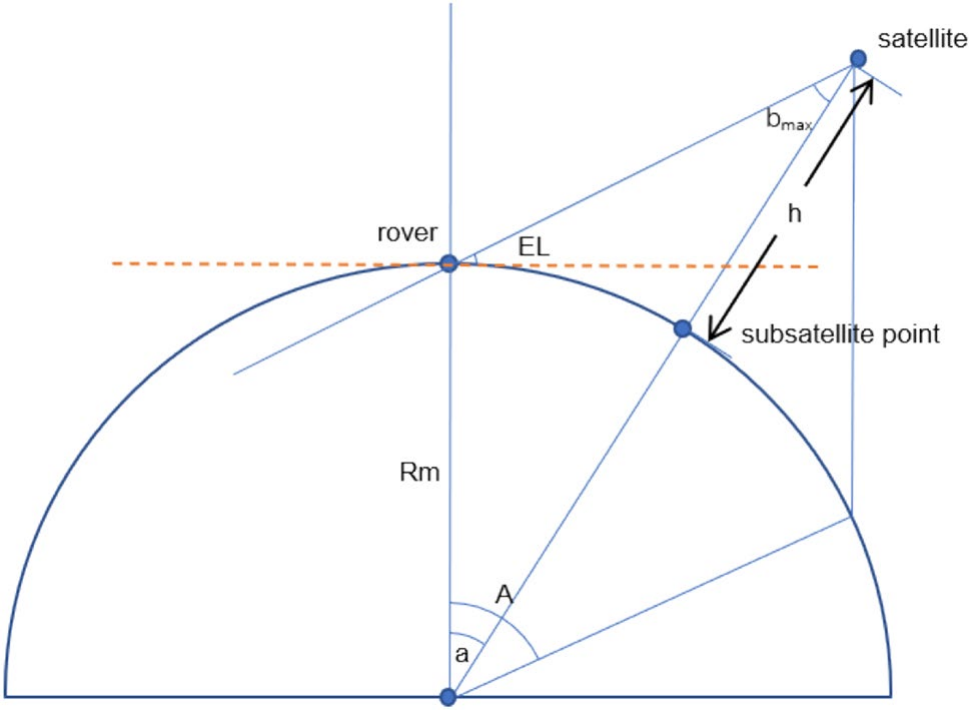
The coverage relationship of a single satellite to the Moon.

To study and analyze the coverage capability of the lunar GNSS satellite to the Moon, the coverage relationship of a single satellite to the Moon is plotted (see Fig. 4).

Where $R_{m}$ is the Moon radius; $EL$ is the satellite altitude angle; $h$ is the satellite orbit altitude; $A$ is the Moon-centered angle covered by the satellite; $a$ is half of the Moon-centered angle covered by the satellite; and $b_{\max }$ is the half-power angle of the satellite signal.

For any position on the Moon, the following equation can be obtained from the sine theorem:

1 $$ \frac{Rm}{{\sin b}} = \frac{Rm + h}{{\sin \left( {EL + \frac{\pi }{2}} \right)}} $$

The angle ${\text{b}}$ between the satellite signal and the line between the satellite and the center of the Moon can be described as:

2 $$ b = \arcsin \left( {\frac{Rm}{{Rm + h}}\sin \left( {EL + \frac{\pi }{2}} \right)} \right) $$

The angle $b$ and the satellite altitude angle can be used to determine whether the current position is within the coverage range of the satellite, which needs to satisfy the following conditions:

3 $$ \left. \begin{aligned} & el > 0 \\ & b < b_{\max } \\ \end{aligned} \right\} $$

As the Moon has no atmosphere and is close to a vacuum, observations of satellite signals have less of an effect caused by the satellite altitude angle factor than they do in the environment of Earth. No cut-off angle of the lunar GNSS satellite altitude is set, i.e. the satellite is considered available when the satellite altitude angle is greater than 0.

To facilitate the calculation, we divide the Moon into a grid with 1° interval by latitude and longitude, and use the 3D coordinate points of the 3D coordinate system with the center of the Moon to represent the 3D coordinates of each grid point. The three-dimensional coordinate system of the center of the Moon is defined x-axis from the Earth pointing to the Moon, the z-axis is perpendicular to the Moon's orbital plane, and the y-axis forms a right-handed coordinate system with the x-axis and z-axis. Latitude and longitude are defined as the line surface angle formed by the line connecting a point on the lunar surface to the lunar center and the x–y plane of the lunar three-dimensional coordinate system, and the line surface angle formed by the line and the x–z axis plane. The arctic region is defined as $latitude \in [60^{ \circ } ,90^{ \circ } ]$, the south pole region is defined as $latitude \in [ - 90^{ \circ } , - 60^{ \circ } ]$, the equatorial region is defined as $latitude \in [ - 60^{ \circ } ,60^{ \circ } ]$.

The three-dimensional coordinates of the grid point are calculated by the formula:

4 $$ \left. \begin{aligned} x & = Rm*\cos (latitude)*\cos (longitude) \\ y & = Rm*\cos (latitude)*\sin (longitude) \\ z & = Rm*\sin (latitude) \\ \end{aligned} \right\} $$

To analyze the satellite visibility capability of a region during its whole orbital period, the satellite visibility during the orbital period (SVOP) parameter is set. SVOP is the time that a region is covered by satellites during the whole orbital period. When SVOP is equal to 1, it means that the area is continuously covered. SVOP value can be calculated by the formula:

5 $$ SVOP = \frac{{\partial {\text{t}}}}{T} $$

where $\partial {\text{t}}$ is the total satellite available time at the grid point, $T$ is the orbital revisit period.

## Results and discussion

### Analysis of the SVOP values

**Figure 5:**
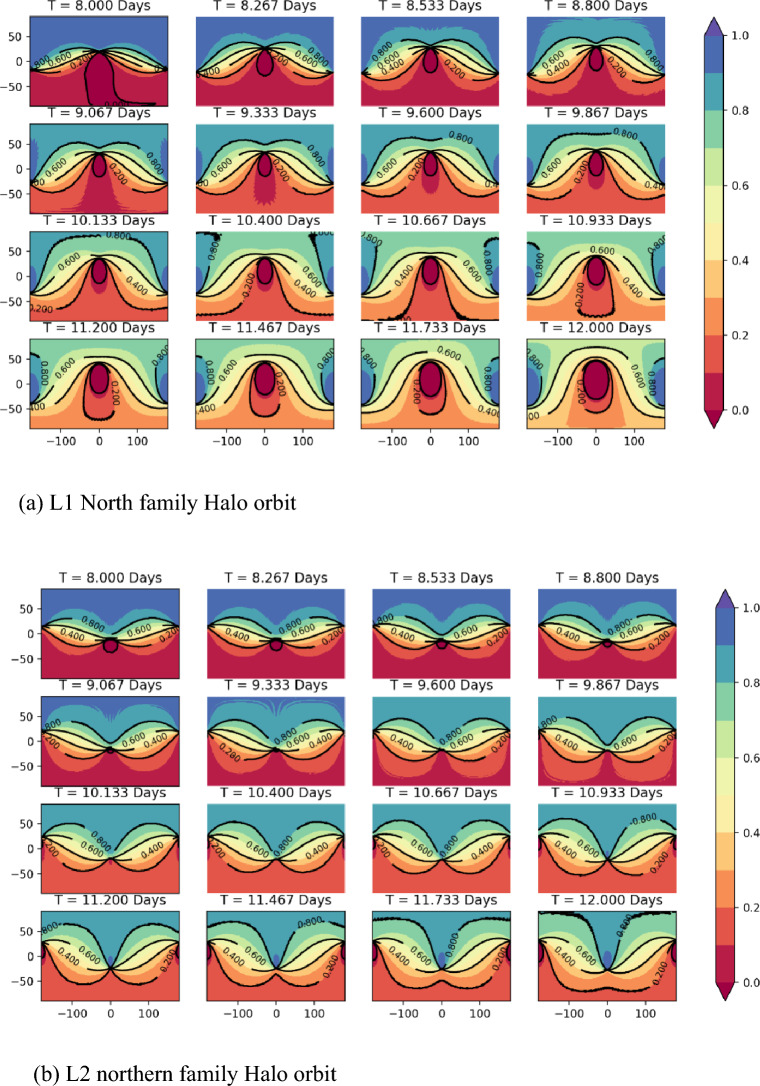
Map of SVOP values for the lunar surface covered by Halo orbit satellites. (**a**) L1 North family Halo orbit. (**b**) L2 northern family Halo orbit.

Referring to the literature^9^, it is known that the period of the L1-point Halo orbit is about 8 to 12 days, the period of the L2-point Halo orbit is between 4 and 15 days, and the period of the DRO is concentrated between 6 and 28 days. To ensure the stability of the multi-orbital constellation, the period of the Halo orbits we study is limited between 8 and 12 days. The simulation calculation results of SVOP values for satellite coverage in Halo orbit are shown in Fig. 5. Where T stands for the orbital period, and the orbital period interval between each picture in the order is about 0.265 days; the colour value stands for the SVOP value; the horizontal coordinate stands for the lunar longitude and the vertical coordinate stands for the lunar latitude (the simulation results of the Halo southern family orbit are omitted in the figure due to the Halo orbit symmetry).

From Fig. 5, it can be seen that satellites operating in Halo-North family orbit with an orbital period of about 8 days have better coverage of the Moon's north polar region with SVOP values above 0.9. This means that most of the northern region is covered well by the satellite more than 90% time of its whole period. As the orbital period gradually increases, i.e. the orbit gradually approaches the LP, the coverage ability of Halo orbit becomes weaker. The simulation results of the Halo southern family orbit are the same as the results of the northern family Halo orbit due to the Halo orbit symmetry.

**Figure 6:**
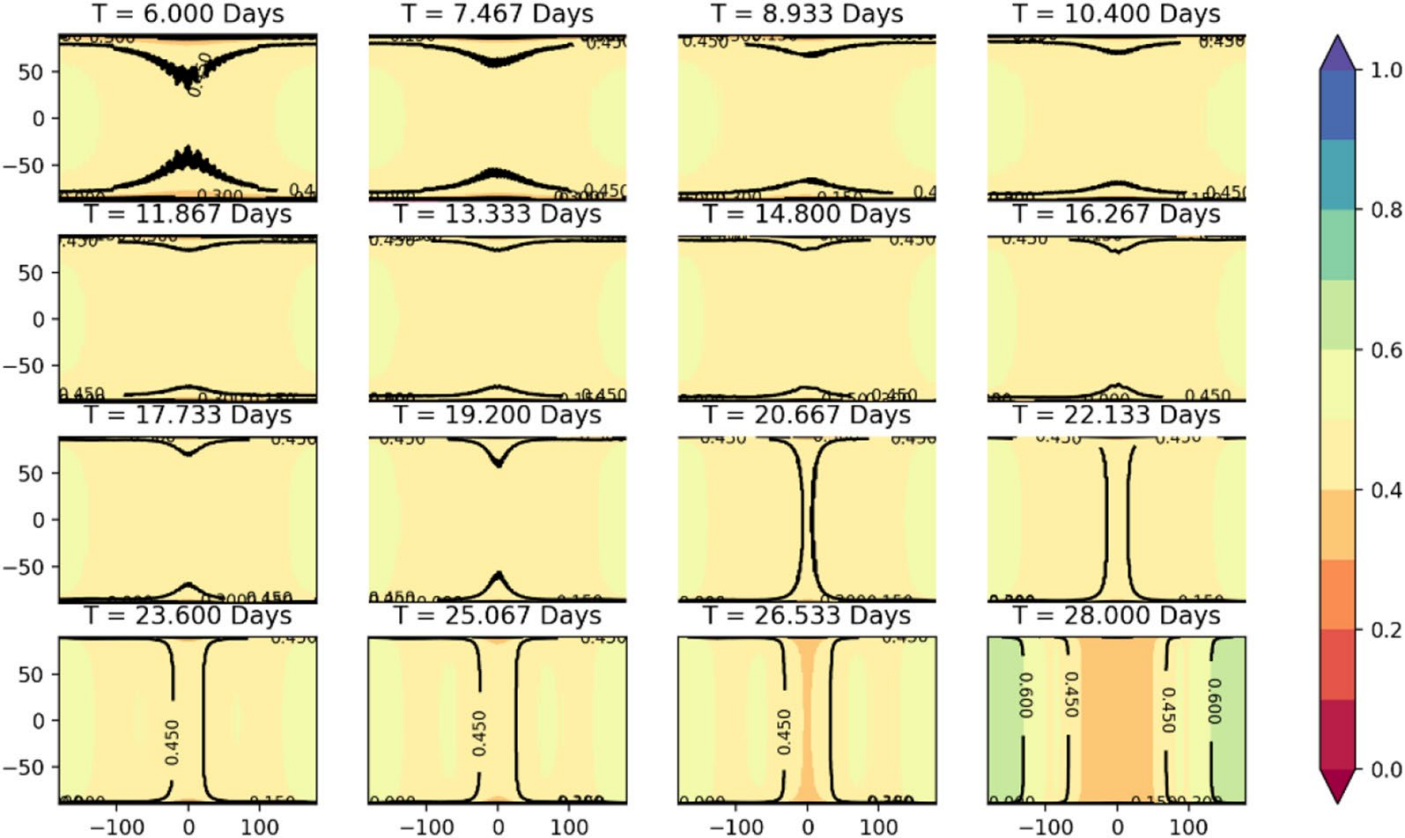
Map of SVOP values for the lunar surface covered by DRO satellites.

The simulation calculation results of SVOP values for satellite coverage in DRO orbit are shown in Fig. 6. Where T stands for the orbital period, and the orbital period interval between each picture in the order is about 1.465 days; the colour value stands for the SVOP value; the horizontal coordinate stands for the lunar longitude and the vertical coordinate stands for the lunar latitude.

As can be seen from Fig. 6, the SVOP values are relatively similar for the whole lunar surface covered by the DRO satellite, around 0.45. The stability of DRO satellites to cover the equatorial region is stronger than that of Halo orbit satellites. When the orbital period is 14.8 days and 16.267 days, the coverage effect of the DRO satellite on the entire lunar surface is the best. However, when the orbital period is around 27 days, the coverage effect of the DRO satellite on the lunar surface also changes due to the variation in the orbital shape.

### Analysis of the minimum number of visible satellites

According to the results of the analysis in "Analysis of the SVOP values" section, the Halo orbits with revisit periods of 8 days and the DRO with revisit periods of 14.680 days and 16.17 days provide the best coverage, so later designs of the lunar GNSS multi-orbit constellation are based on these orbits. The cost of inserting a satellite into orbit around the L2 point is about 3.2018 KM/S and the cost of keeping satellite in orbit for one year is about 102.3 M/S^22^. With experience from GNSS constellation design, we first study the case of 3 orbits and then gradually expand to 4, 5 and more orbits. For various multi-orbital lunar GNSS constellations, the minimum number of visible satellites for each grid point in an orbital period is plotted. If the minimum number of visible stars for each grid point is greater than 4, the constellation design meets the requirement of providing real-time PNT services.

L1N, L1S, L2N, L2S, DRO1 and DRO2 are used in each case to represent the L1 point northern halo orbit with a period of 8 days, the L1 point southern halo orbit with a period of 8 days, the L2 point northern halo orbit with a period of 8 days, the L2 point southern halo orbit with a period of 8 days and the DRO with a period of 14.680 days and the DRO with a period of 16.17 days, respectively.

1. The Case of 3 orbits
  To satisfy the full coverage of the Moon's surface, considering the constellation design of GNSS, a 3-orbital constellation design, which consist of two Halo orbits of different families and one DRO, is discussed here. According to the Earth GNSS positioning theory, the minimum number of visible satellites to meet the positioning requirements is 4. If there are only 3 orbits to form the lunar GNSS constellation, then those three orbits must include one Halo southern family orbit, one Halo northern family orbit, and one DRO. Based on SVOP analysis results, we can conclude that the DRO and Halo orbits cannot provide continuous coverage of certain area of the Moon. If only 4 satellites are deployed in each of the three orbits, it will not meet the positioning requirements. Therefore, we choose to deploy five satellites in each orbit. Combining with the satellite composition and star selection theory of GNSS^23^, it is observed that the better the spatial distribution of satellites when performing satellite positioning, the better the positioning effect. The satellites of one orbit are evenly distributed, phase difference of the adjacent satellites is ${{2*\pi } \mathord{\left/ {\vphantom {{2*\pi } 5}} \right. \kern-0pt} 5}$.
  All possible cases are shown in Table 1. Due to the symmetry of the Halo orbit, where Case 3-3 and Case 3-4 are symmetric, we only need to discuss 3 cases. The results are shown in Fig. 7.

**Table 1:** The Case of 3 orbits.

Orbit	L1N	L1S	L2N	L2S	DRO1
Case 3-1	5	5	–	–	5
Case 3-2	–	–	5	5	5
Case 3-3	5	–	–	5	5
Case 3-4	–	5	5	–	5

**Figure 7:**
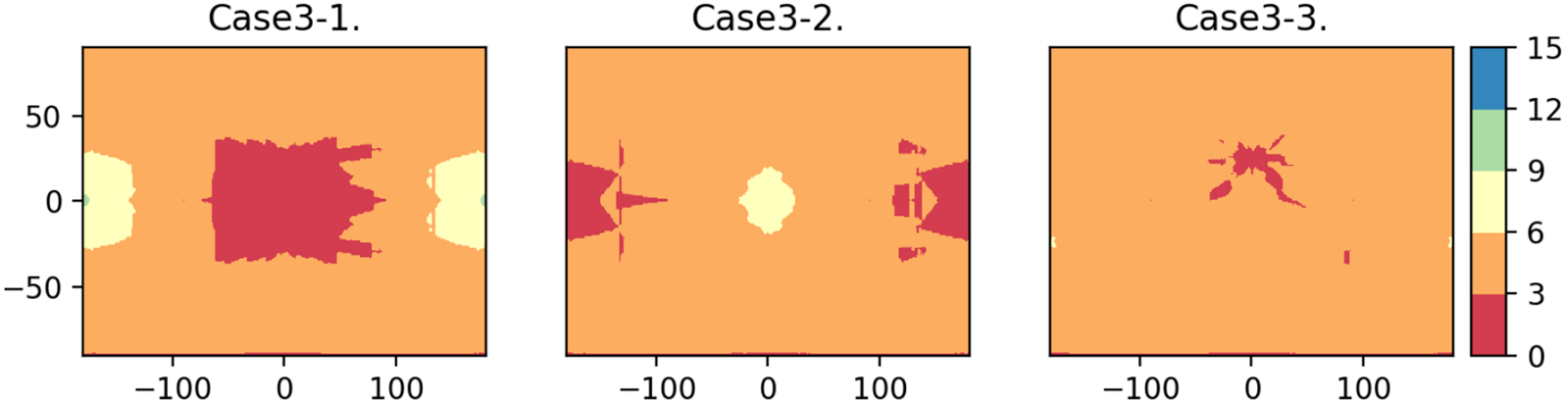
Minimum number of visible satellites on the lunar surface for the case of 3 orbits.
  In the figure, the red part indicates that the minimum number of visible satellites is between 0 and 3, which means that the satellite positioning requirements are not met. It can be seen in Fig. 7 that all three types of tri-orbit lunar GNSS constellations have some areas that do not meet the satellite positioning requirements. Particularly, Case 3-1 and Case 3-2 show gaps in coverage for equatorial regions, where some areas only have one visible satellite sometimes, whereas Case 3-3 exhibits a slightly improved coverage capability compared to Case 3-1 and Case 3-2. Hence, a three-orbital lunar GNSS constellation comprising fifteen satellites fails to meet the requirement of real-time positioning across the entire lunar surface.
2. The Case of 4 orbits
  Based on the simulation experiment results of the three-orbit lunar GNSS constellation and considering the number of orbit strips and the number of satellites, this subsection will discuss the case of adding one orbit. One or two DROs must be included in the orbit combinations to achieve better full lunar surface coverage. Let there be four satellites in each orbit, and the phase difference of the adjacent satellites is ${\pi \mathord{\left/ {\vphantom {\pi 2}} \right. \kern-0pt} 2}$. All possible cases are shown in Table 2. Due to the symmetry of Halo orbits, Case 4-1 and Case 4-2, Case 4-3 and Case 4-4, and Case 4-6 and Case 4-8 are symmetric, it is sufficient to analyze only five cases, i.e. Case 4-1 and Case 4-2, Case 4-3 and Case 4-4, and Case 4-6 and Case 4-8. The results of 4-orbital constellation are shown in Fig. 8.

**Table 2:** The Case of 4 orbits.

Orbit	L1N	L1S	L2N	L2S	DRO1	DRO2
Case 4-1	4	4	4	–	4	–
Case 4-2	4	4	–	4	4	–
Case 4-3	4	–	4	4	4	–
Case 4-4	–	4	4	4	4	–
Case 4-5	4	4	–	–	4	4
Case 4-6	4	–	–	4	4	4
Case 4-7	–	–	4	4	4	4
Case 4-8	–	4	4	–	4	4

**Figure 8:**
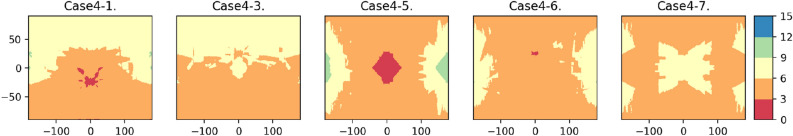
Minimum number of visible satellites on the lunar surface for the case of 4 orbits.
  From Fig. 8, we can see that both Case 4-3 and Case 4-7 demonstrate the coverage capability to meet the condition of the minimum number of the visible satellites for navigation and positioning. Of particular note is that Case 4-5 represents a 4-orbital constellation composed of two Halo orbits located at the L1-point with two Distant Retrograde Orbits (DROs). Case 4-7 is a 4-orbital constellation consisting of two Halo orbits located at the L2-point with two DROs. There is a clear contrast in the equatorial region between the two cases. Therefore, the equatorial coverage capability of L2-point Halo orbit satellites is prior to L1-point Halo orbit counterparts. Thus, in multi-orbital constellation design, L2-point Halo orbit is recommended.
3. The Case of more than 5 orbits
  According to the analysis results of the previous three and four orbits, there is a constellation with 4 orbits and 16 satellites that meets the positioning conditions. Therefore, in the case of 5 orbits and more, the number of satellites cannot exceed 16. Considering the launch and maintenance costs of multi-orbit satellites, on the basis of the analysis results with the previous two subsections, equal or less than 6 orbits and about 15 satellites are recommended in the multi-orbit lunar GNSS constellation.

**Figure 9:**
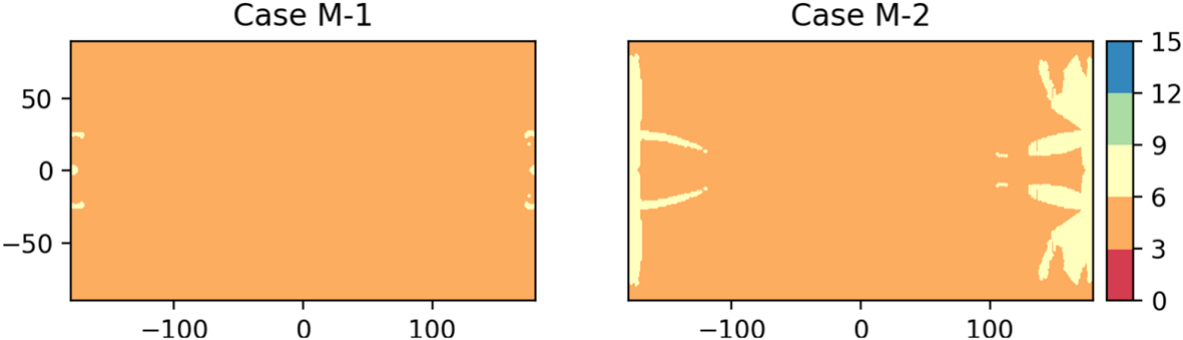
Minimum number of visible satellites on the lunar surface for the case of more than 5 orbits.

Considering the number of orbits and satellites, the following two cases with better coverage performance are given (see Table 3). Both cases can meet the requirements of real-time positioning with 14 and 16 satellites, respectively, while the diversity of orbit types can ensure a better satellite geometry distribution, and their minimum visible satellite number maps are shown in Fig. 9.

**Table 3:** The Case of more than 5 orbits.

Orbit	L1N	L1S	L2N	L2S	DRO1	DRO2
Case M-1	2	2	2	2	6	–
Case M-2	2	2	2	2	4	4

### Analysis of multi-orbit lunar GNSS constellation positioning capability

As mentioned above, there exist four sets of multi-orbit constellation design schemes that can be meet the requirement of the minimum visible satellite number condition for lunar GNSS positioning. These schemes are identified as Case 4-3, Case 4-7, Case M-1 and Case M-2 (Table 4). To analyze the navigation and positioning effects of these four multi-orbital constellation design solutions across different regions of the Moon, an accuracy evaluation model has been constructed later.

**Table 4:** Multi-orbit lunar GNSS constellation designs that meet positioning requirements.

Orbit	L1N	L1S	L2N	L2S	DRO1	DRO2
Case M-1	2	2	2	2	6	–
Case M-2	2	2	2	2	4	4
Case4-3	4	–	4	4	4	–
Case4-7	–	–	4	4	4	4

### Accuracy evaluation model

The main influencing factors of positioning accuracy are the ranging accuracy and the geometric distribution of the visible satellites. The geometric distribution of satellites can be expressed by the Dilution of Precision (DOP) factor, and then the positioning accuracy can be expressed as:

6 $$ \sigma = DOP \cdot \sigma_{0} $$

where $\sigma$ is the positioning accuracy; $\sigma_{0}$ is the observation accuracy. To calculate the DOP value, the Jacobi matrix $A$ is required, which can be described as:

7 $$ {\mathbf{A}} = \left[ {\begin{array}{*{20}c} {\frac{{x^{S} - x_{R} }}{{\rho_{R}^{S} }}} & {\frac{{y^{S} - y_{R} }}{{\rho_{R}^{S} }}} & {\frac{{z^{S} - z_{R} }}{{\rho_{R}^{S} }}} & { - C} \\ {\frac{{x^{S} - x_{R} }}{{\rho_{R}^{S} }}} & {\frac{{y^{S} - y_{R} }}{{\rho_{R}^{S} }}} & {\frac{{z^{S} - z_{R} }}{{\rho_{R}^{S} }}} & { - C} \\ {\frac{{x^{S} - x_{R} }}{{\rho_{R}^{S} }}} & {\frac{{y^{S} - y_{R} }}{{\rho_{R}^{S} }}} & {\frac{{z^{S} - z_{R} }}{{\rho_{R}^{S} }}} & { - C} \\ {\frac{{x^{S} - x_{R} }}{{\rho_{R}^{S} }}} & {\frac{{y^{S} - y_{R} }}{{\rho_{R}^{S} }}} & {\frac{{z^{S} - z_{R} }}{{\rho_{R}^{S} }}} & { - C} \\ \end{array} } \right] $$

where $x^{S}$, $y^{S}$, $z^{S}$ are the three-dimensional coordinates $x$, $y$, $z$ of the satellite in the Earth–Moon center of mass convergence coordinate system, and $x_{R}$, $y_{R}$, $z_{R}$ are the three-dimensional coordinates $x$, $y$, $z$ of the receiver in the Earth–Moon center of mass convergence coordinate system, respectively. $C$ is the speed of light. The matrix ${\mathbf{Q}}$ can be calculated from the Jacobi matrix ${\mathbf{A}}$, which is given by the following formula:

8 $$ \begin{aligned} {\varvec{Q}} & = ({\varvec{A}}^{{\text{T}}} {\varvec{A}})^{ - 1} \\ {\varvec{Q}} & = \left[ {\begin{array}{*{20}c} {\sigma_{x}^{2} } & {\sigma_{xy}^{2} } & {\sigma_{xz}^{2} } & {\sigma_{xt}^{2} } \\ {\sigma_{xy}^{2} } & {\sigma_{y}^{2} } & {\sigma_{yz}^{2} } & {\sigma_{yz}^{2} } \\ {\sigma_{xz}^{2} } & {\sigma_{yz}^{2} } & {\sigma_{z}^{2} } & {\sigma_{zt}^{2} } \\ {\sigma_{xt}^{2} } & {\sigma_{yt}^{2} } & {\sigma_{zt}^{2} } & {\sigma_{t}^{2} } \\ \end{array} } \right] \\ \end{aligned} $$

where, $\sigma$ is the standard deviation. DOP values are also divided into various types, including Time Dilution of Precision (TDOP), Horizontal Dilution of Precision (HDOP), Position Dilution of Precision (PDOP) and Geometric Dilution of Precision (GDOP), which indicate the precision strength of the parameter, respectively. Their values can be described as:

9 $$ \left. \begin{aligned} TDOP & = \sqrt {\sigma_{t}^{2} } \\ HDOP & = \sqrt {\sigma_{x}^{2} + \sigma_{y}^{2} } \\ PDOP & = \sqrt {\sigma_{x}^{2} + \sigma_{y}^{2} + \sigma_{z}^{2} } \\ GDOP & = \sqrt {\sigma_{x}^{2} + \sigma_{y}^{2} + \sigma_{z}^{2} + \sigma_{t}^{2} } \\ \end{aligned} \right\} $$

Here the PDOP value is adopted to calculate positioning accuracy, as it can effectively reflect the geometric distribution of satellites. A smaller PDOP value indicates a better satellite distribution geometry, i.e. the positioning accuracy is higher. Conversely, a larger PDOP value results stand for poorer accuracy.

### Simulation results

**Figure 10:**
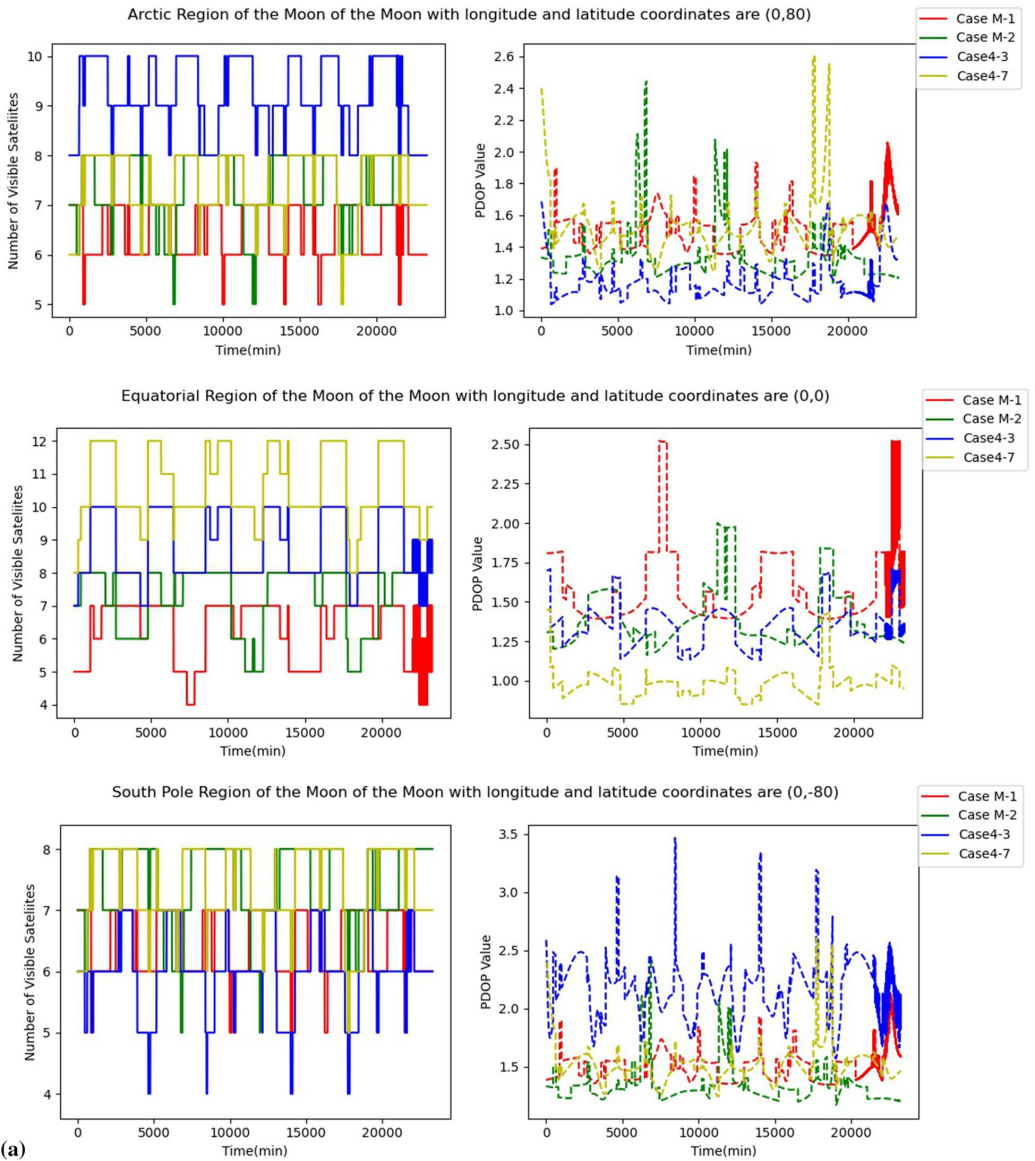

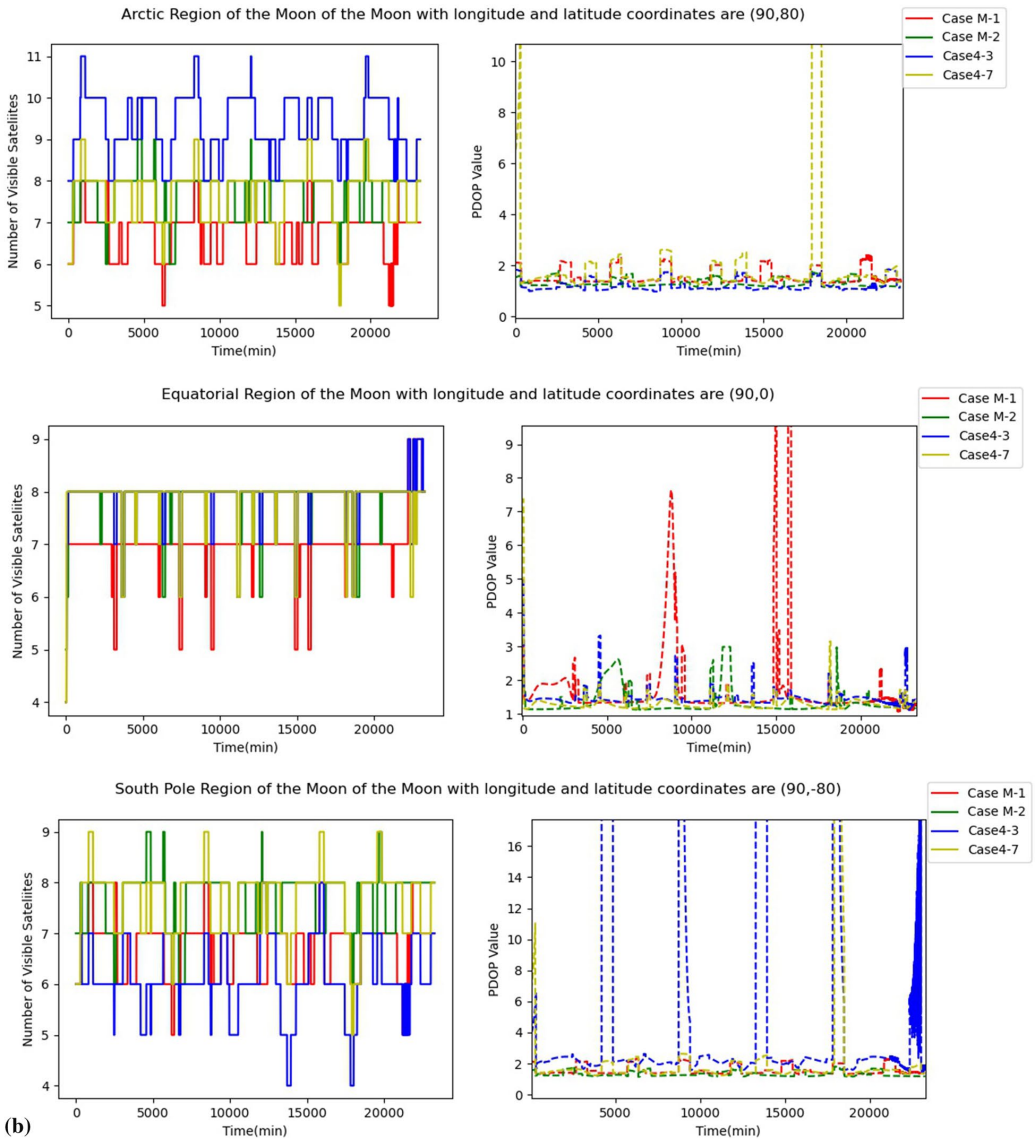
Number of visible satellite and PDOP values for different regions of the multi-orbital lunar GNSS constellation design. (**a**) Grid points in the three regions of the Moon with longitude 0. (**b**) Grid points in the three regions of the Moon with longitude 90.

Two grid points each at the Moon's South Pole, Arctic and Equator were chosen to investigate the navigation and positioning effects of four multi-orbital lunar GNSS constellation designs at different longitudes and latitudes. We carried out simulations of the four multi-orbital lunar GNSS constellations for 16.17 days to calculate the number of the visible satellites and PDOP values of the grid points. The results of the simulation experiment were then plotted for each grid point, as shown in Fig. 10.

As can be seen in Fig. 10, the number of visible satellites of the four constellations in the three regions of the Moon ranges from 4 to 12. In Fig. 10a, the PDOP values of the four constellations at the same longitude with different latitudes do not have a large difference and are stable between 1.0 and 3.0. However, in Fig. 10b, which has the same latitude and different longitude as Fig. 10a, the PDOP values of Case M-1, Case4-3 and Case4-7 all undergo a surge at some epochs. This phenomenon indicates the longitude dependence of PDOP values.

To sum up, Cases M-2 demonstrate relatively stable performance, with the PDOP values remain low at different longitudes and latitudes. Considering the performance of Case M-2, they can be used to build a lunar GNSS constellation to provide real-time PNT services for the Moon activities.

## Conclusions

Navigation and positioning are very important to survey and explore the Moon. Multi-orbital lunar GNSS constellations composing of Halo orbit and DRO are studied and analysed in this paper. The main conclusions can be summarized as follows:

1. Halo orbit and the DRO are related with libration points. The satellites in these orbits have good coverage effect to the moon. The Halo southern or northern family orbit with an 8-day period demonstrates superior coverage effectiveness in the South Pole or Arctic regions of the Moon. The coverage effect of the DRO was relatively stable, with an SVOP value of more than 0.45 in the equatorial region of the moon.
2. Multi-orbital lunar GNSS constellation design combining Halo orbit and DRO is studied by simulation experiment and theoretical analysis. And one constellation design scheme with six orbits and 16 satellites is selected.
3. Based on the selection schemes about multi-orbital lunar GNSS constellation, accuracy evaluated model are given. The simulation experimental results shown that a bigger number of the visible satellites and a smaller PDOP value within 2.0 in the most time in the Moon surface regions.

This study offers insightful and constructive ideas for the future establishment of a lunar GNSS. The multi-orbital constellation design schemes have the potential and practical value for the lunar navigation and positioning applications.

## Supplementary Information

Supplementary Information.

## Data Availability

The authors confirm that the data supporting the findings of this study are available within the article and its supplementary materials. The supplementary materials include simulation data from the experiments and details on how to obtain them are provided in the file description document. The ephemeris files for the libration orbits analyzed in this study can be downloaded from the Jet Propulsion Laboratory's Small-Body Orbit Database, website link: https://ssd.jpl.nasa.gov/tools/periodic_orbits.html.

## References

[1] Gil A, David A, Renwick D, Cappelletti C, Blunt P (2023). Methodology for optimizing a constellation of a lunar global navigation system with a multi-objective optimization algorithm. Acta Astronaut..

[2] Li X, Cheng F, Zhao H, Shen P, Liu D (2022). Analysis on visibility and signal strength of satellite for lunar navigation. Sci. Surv. Mapp..

[3] Bin LIU, Xiyun HOU, Jingshi TANG, Lin LIU (2017). Autonomous orbit determination of satellites around triangular libration points in the Earth–Moon system. J. Spacecr. TT&C Technol..

[4] Yun-He M, Qi-Feng C (2014). Outline design & performance analysis of navigation constellation near Earth–Moon libration point. Acta Phys. Sin..

[5] *China National Space Administration*. http://www.cnsa.gov.cn/n6758823/n6758838/c6801903/content.html (2018).

[6] Farquhar RW (1967). Lunar communications with libration-point satellite. J. Spacecr. Rocket.

[7] Carpenter, J. R. *et al*. Libration point navigation concept supporting the vision for space exploration. In* AIAA/AAS Astrodynamics Specialist Conference* (2004).

[8] Wu W, Tang Y, Zhang L, Qiao D (2018). Design of communication relay mission for supporting lunar-farside soft landing. Sci. China (Inf. Sci.).

[9] Gao Z-Y, Hou X-Y (2020). Coverage analysis of lunar communication/navigation constellations based on Halo orbits and distant retrograde orbits. J. Navig..

[10] Hamera, K. *et al.* An evolvable lunar communication and navigation constellation concept. In *2008 IEEE Aerospace Conference.* (2008).

[11] Batista, A., Gomez, E., Qiao, H. & Schubert, K. E. Constellation design of a lunar global positioning system using cubesats and chip-scale atomic clocks. In *WorldComp 2012 Proceeding – Embedded Systems and Applicatioin* (2012).

[12] Circi C, Romagnoli D, Fumenti F (2014). Halo orbit dynamics and properties for a linar global positioning system design. Mon. Not. R. Astron. Soc..

[13] Zhang L, Bo Xu (2016). Simplified constellation architecture for the libration point satellite navigation system. J. Navig..

[14] Pereira, F. & Selva, D. Exploring the design space of lunar GNSS in frozen orbit conditions. In* 2020 IEEE/ION Position, Location and Navigation Symposium (PLANS)*. (2020).

[15] Pasquale A (2022). Cislunar distributed architectures for communication and navigation services of lunar assets. Acta Astronaut..

[16] Zanotti G (2022). High performance lunar constellation for nacigation services to Moon orbiting users. Adv. Space Res..

[17] Nallapu, R., Vance, L. D., Xu, Y. & Thangavelatham, J. Automated design architecture for lunar constellations. In* 2020 IEEE Aerospace Conference* (2020).

[18] Zhou J, Hu J, Zhang B (2020). Approximate analytical solutions of motion near the collinear libration-points in restricted three-body problem. J. Deep Space Explor..

[19] Ping S, Guangji QU (2005). Time synchronization techniques of the autonomous navigation of navigation constellation. Chin. J. Astronaut..

[20] Wu, G., Wang, J., Zhang, R. & Wang. Y. Halo orbit satellite orbiting and simulation calculation. In *Proceedings of the 3rd Academic Conference of the Deep Space Exploration Technology Committee of the Chinese Academy of Astronautics*. (2006).

[21] Farquhar RW, Dunham DW, Guo Y, McAdams JV (2004). Utilization of libration points for human exploration in the Sun–Earth–Moon system and beyond. Acta Astronaut..

[22] Xu M, Wang J, Liu S, Xu S (2013). A new constellation configuration scheme for communicating architecture in cislunar space. Math. Probl. Eng..

[23] Shi JP (2023). Fast satellite selection algorithm for GNSS multi-system based on Sherman–Morrison formula. GPS Solut..

