# Supplementary material for: Multi-orbit lunar GNSS constellation design with distant retrograde orbit and Halo orbit combination
Source: Sci Rep. 2023 Jun 22;13:10158. doi: 10.1038/s41598-023-37348-x (PMC10287729; doi:10.1038/s41598-023-37348-x)
Supplement: Supplementary file 1 — Supplementary Information. [file 41598_2023_37348_MOESM1_ESM.zip › supplement materials of the manuscript/document description.docx]

**Data Description**

The detail and the format of the data about Figure 3, Figure 5-10 are described as follows,

**Figure 3**

The "Figure 3" folder contains all the data for Figure 3. The "Figure 3" folder contains the "Halo Orbit" folder and the "Distant Retrograde Orbit" folder. The "Halo Orbit" folder and the "Distant Retrograde Orbit" folder contain the satellite position files for Halo orbit and DRO respectively. The "Halo Orbit" folder contains "L1N", "L1S", "L2N" and "L2S", which store the satellite position files for the Halo orbits of the L1 point North, L1 point South, L2 point North and L2 point South families, respectively. The satellite position files can be obtained from the JPL Small-Body Database of the Jet Propulsion Laboratory, a division of NASA, and downloaded athttps://ssd.jpl.nasa.gov/tools/periodic_orbits.html.The satellite position file type is a CSV file, and the first line indicates the data format, as shown in the following table:

| Time (TU) | X (LU) | Y (LU) | Z (LU) | VX (LU/TU) | VY (LU/TU) | VZ (LU/TU) |
| --- | --- | --- | --- | --- | --- | --- |

where TU is the normalized time unit, denoting 382981s, and LU is the normalized length unit, denoting 389703km. "Time(TU)" denotes the satellite operation time; "X(LU)", "Y(LU)", "Z(LU)" indicate the three-dimensional coordinates of satellite operation; "VX(LU/TU)", "VY(LU/ TU)", "VZ(LU/TU)" indicate the satellite orbital speed.

**Figure 5**

The "Figure 5" folder contains all the data for Figure 5. The "Figure 5" folder contains the "L1 North Family Halo orbit" folder and the "L2 North Family Halo orbit". "L1 North Family Halo orbit" folder and the "L2 North Family Halo orbit" folder contain the satellite position files and the SVOP calculation result files for the L1 North Family and L2 North Family Halo orbit satellites, respectively. Both the "L1 North Family Halo orbit" folder and the "L2 North Family Halo orbit" folder contain the satellite position files and the " SVOP" folder, and the "SVOP" folder contains the SVOP value calculation result files. The data format of the satellite position file is the same as that of Figure 3. The SVOP value calculation result file is a CSV file with 180 rows and 360 columns, the row and column numbers represent the latitude and longitude of the lunar position in the cell, and the value of the cell represents the SVOP value.

**Figure 6**

The "Figure 6" folder contains all the data for Figure 6. The data is stored in the same file format as Figure 5.

**Figure 7**

The "Figure 7" folder contains all the data for Figure 7. The "Figure 7" folder contains the "Case 3-1" folder, the "Case 3-2" folder, and the "Case 3-3" folders, which contain the satellite position files and the minimum number of the visible satellites calculation files for Case 3-1, Case 3-2 and Case 3-3, respectively. The data format of the satellite position file is the same as that in Figure 3. The minimum number of visible stars calculation result file is a CSV file, the file name is the name of the folder, there are 180 rows and 360 columns, the row number and column number represent the latitude and longitude of the lunar position in the cell, the value of the cell represents the minimum number of the visible satellites.

**Figure 8**

The "Figure 8" folder contains all the data for Figure 8. The "Figure 8" folder contains the "Case 4-1" folder, the "Case 4-3" folder, the "Case 4-5" folder, "Case 4-6" folder, and "Case 4-7" folder, which including the satellite position files for Case 4-1, Case 4-3, Case 4-5, Case 4-6, and Case 4-7 satellite position files and minimum visible stars calculation result files. The file format of the satellite position file and the minimum number of the visible satellites file is the same as that of Figure 7.

**Figure 9**

The "Figure 9" folder contains all the data for Figure 9. The "Figure 9" folder contains the "Case M-1" folder and the "Case M-2" folder, which including the satellite position files and the minimum number of the visible satellites calculation files for Case M-1 and Case M-2, respectively. The "Case M-1" folder and the "Case M-2" folder contain the satellite position files and the minimum number of the visible satellites calculation result files for Case M-1 and Case M-2, respectively. The file format of the satellite position file and the minimum number of the visible satellites file is the same as Figure 7.

**Figure 10**

The "Figure 10" folder contains all the data for Figure 10. The "Figure 10" folder contains the "Arctic Region of the Moon" folder, the "Equatorial Region of the Moon" folder, and the "South Pole Region of the Moon" folder. "Arctic Region of the Moon" folder, "Equatorial Region of the Moon" folder, "South Pole Region of the Moon" folder. Each folder includes the "Case 4-3" folder, " Case 4-7" folder, "Case M-1" folder, and "Case M-2" folder, which including the file of the Arctic Region of the Moon, the Equatorial Region of the Moon, and the South Pole Region of the Moon, respectively. The files of PDOP value and the number of the visible satellites about the multi-orbit lunar GNSS constellation design scenarios are named "PDOP Value" and "Number of Visible Satellites" respectively. And the file type is CSV file with one column, which indicates the PDOP value calculation result or the number of the visible satellites calculation result of the constellation.
